# Proximal Aortic Neck Progression: Before and After Abdominal Aortic Aneurysm Treatment

**DOI:** 10.3389/fsurg.2017.00023

**Published:** 2017-05-04

**Authors:** Konstantinos A. Filis, George Galyfos, Fragiska Sigala, Konstantinos Tsioufis, Ioannis Tsagos, Georgios Karantzikos, Christos Bakoyiannis, George Zografos

**Affiliations:** ^1^First Department of Propedeutic Surgery, Ippokrateion Hospital, School of Medicine, National and Kapodistrian University of Athens, Athens, Greece; ^2^First Department of Cardiology, Ippokrateion Hospital, School of Medicine, National and Kapodistrian University of Athens, Athens, Greece; ^3^First Department of Surgery, Laikon Hospital, School of Medicine, National and Kapodistrian University of Athens, Athens, Greece

**Keywords:** aortic neck, progression, dilatation, open repair, endovascular repair

## Abstract

Several risk factors including short or highly angulated proximal aortic neck have been associated with long-term outcomes after endovascular or open abdominal aortic aneurysm (AAA) repair. However, research data have emerged recently concerning the behavior of proximal aortic neck, and several authors have tried to evaluate this behavior after endovascular or open repair. Additionally, computed tomography angiography (CTA) remains the golden standard for detecting and observing the morphology of an AAA, both before and after treatment. Moreover, the question of whether the proximal neck’s progression independently affects postoperative morbidity and reintervention risks still remains. Therefore, this focused review aims to present all relevant data on the behavior of an AAAs neck, based on CTA imaging before and after repair, in order to produce useful conclusions for future clinical practice.

## Introduction

Both open and endovascular repair have been proved to be effective treatments for patients with abdominal aortic aneurysm (AAA) of significant size (diameter over 5.5 cm) (1). However, when preparing the therapeutic strategy for such patients, careful preoperative assessment of the aortic morphology using computed tomography angiography (CTA) imaging is mandatory. The proximal and distal landing zones are especially relevant to achieve a durable result (2). Although neck size and morphology are major factors that significantly affect the selection of therapeutic method, an increasing number of AAAs with unfavorable proximal neck anatomy are currently treated with standard endograft devices (3).

However, emerging literature data seem to reveal that the behavior of proximal aortic neck plays a significant role on major outcomes after endovascular or open AAA surgery (4). Both endovascular and conventional repair of AAAs with difficult anatomy have been associated with significant long-term morbidity including endoleak or migration, and para-anastomotic aneurysms, respectively (5, 6). The evolution of proximal neck after treatment could be associated with late complications, affecting both long-term morbidity and mortality. Therefore, aim of this review is to collect and present data on the behavior of AAA proximal neck, based on CTA findings before and after treatment, in order to produce useful conclusions for everyday clinical practice.

## Progression of AAA Proximal Neck before Treatment

The aorta exhibits a wide variety of morphologic changes throughout the cardiac cycle. The diameter variation of the proximal aneurysm neck of individual patients ranges from less than 1 to up to 4 mm or more during the cardiac cycle (7). Currently, the modality of choice for preoperative evaluation of AAA is CTA, although magnetic resonance angiography (MRA) can also be used (8). However, in general, the usual CTA protocols acquire static images of the aorta, which, with the current high-speed CT acquisition times, could be at any random moment during the cardiac cycle. Therefore, this could imply inaccurate estimation of aorta’s characteristics, affecting the selection of the proper graft and perhaps the outcome (9). Moreover, data indicate that significant changes per heartbeat are reported in the AAA neck and thoracic aorta as well (10).

The natural history of the aneurysm neck is one of expansion and shortening that will not affect most patients under surveillance. Patients with marginal neck lengths (range: 15–20 mm) at the initial CTA imaging are more likely to experience loss of neck length that may negatively affect endovascular suitability. Specifically, some authors have shown that aneurysm neck diameter increases at an average 0.26 mm and length decreases an average of 1 mm each year during surveillance (11). Especially, during periods of observation, a small but finite percentage of patients (8%) will experience a decrease in length to <15 mm, but those with marginal neck lengths of 15–20 mm have increased chance (36%) of shortening <15 mm (11). Finally, in the subgroup of patients with small AAAs, a recent meta-analysis has shown that mean AAA diameter growth rates ranged from 2.6 to 5.2 mm/year (12). Factors reported to be associated with increased AAA expansion in this study included: large AAA thrombus size (*n* = 3 studies), large baseline AAA diameter (*n* = 2 studies), high AAA wall stress, elevated plasma concentration of matrix metalloproteinase-9 (MMP-9), and presence of carotid artery disease (*n* = 1 study each) (12). The same factors could play a significant role in AAA neck expansion as well.

Regarding pathophysiology, the role of immunity in AAA progression is controversial. There are studies suggesting that immune pathways are also upregulated within the non-dilated aorta proximally to an AAA, with over a thousand differentially expressed genes detected in AAA necks (13). The seemingly non-diseased infrarenal AAA neck in patients with AAA undergoing surgical repair shows histological signs of destruction and upregulation of potential drug targets. Kaladji et al. have found that the dilatation of the proximal neck seems to homogenously affect the entire area of the neck rather than just the zone immediately below the renal arteries (14). Moreover, an increased aneurysmal burden as expressed by large aortic neck diameters and AAA size has been shown to be an independent risk factor for continuing aortic neck dilatation (15). Specifically, aneurysm expansion has been correlated with the density of the inflammatory cells, which in turn increases MMP activity and leads to compromised wall strength. This biologic activity is localized, such that spots of increased expression and activation of MMPs might contribute to fast local aneurysm expansion (16). However, we have shown recently that osteopontin—a novel inflammation marker—is associated with AAA presence but not AAA extent, although osteoprotegerin is not associated either with aneurysm presence or with aneurysm extent (17). These data indicate indirectly that aneurysmal sac composition is different compared to the proximal neck causing probably a different behavior to the wall stress. Experimental data show that at high stresses—such as in hypertensive patients—mostly collagen is involved in carrying the stresses (18). Therefore, since distal abdominal aorta has a higher concentration of collagen fibers compared to the more proximal aorta, there could be differences in their behavior.

Finally, differences in AAA neck morphology are also observed between the two genders. According to a recent study (19), neck angulation was greater in women (23.9° vs. 13.5°; *P* < 0.028), and the percent thrombus in women was higher than men as well (35.4 vs. 31%; *P* < 0.02). Additionally, AAAs were smaller in women at 1 year (4.2 vs. 5.1 cm; *P* < 0.002), and secondary interventions were higher in men in the same study (11.3 vs. 0%; *P* < 0.05). Other features such as neck shape, changes in neck diameter, neck length, and percent oversizing of graft were not statistically different between genders. However, the same authors concluded that these gender differences in neck characteristics and changes in neck morphology do not appear to adversely affect treatment outcomes (19).

## Progression of Proximal AAA Neck after Evar

Several studies have reported a postoperative neck dilatation in 10–36% of cases, with loss of proximal fixation resulting in graft instability and increased risk for migration or endoleak (20–22). An increase of 5.0 mm is considered significant (10% of an average aneurysm size), according to Society of Vascular Surgery (SVS) standards for EVAR (23). However, recent pooled data have shown that the mean (or median) distension 1 cm below the most distal renal artery is at least 2.0 mm, without being significantly different from pre-EVAR values (10). Other authors report an increase of more than 2.0 mm, in more than one-third of patients treated endovascularly (24). This increase is most commonly evident within the first 2 years after EVAR. Soberón et al. underline that this increase occurs specifically within the first 6 months although within the period from 6 to 24 months, no significant neck variation is observed (25). In majority of cases, dilatation of the aneurysm neck does not significantly exceed stent-graft diameter and, therefore, it could be possibly related to the presence of the endograft.

Postoperative surveillance of these cases is most frequently performed with CTA scanning as well as plain film interrogation, with MRI reserved for patients whose renal function will not tolerate iodinated contrast. The decision for reintervention is complex and involves situations such as development of type I or III endoleak, loss of proximal or distal stent-graft fixation, device migration, continued aneurysm expansion with a type II endoleak, or device fatigue with structural failure (26). As aforementioned, the proximal sealing and fixation zone of a stent graft in the aortic neck expands significantly per heartbeat, both before as well as after EVAR. Therefore, maximum diameter using dynamic MRA may not be similar to the maximum diameter with static CTA in all patients, and a standard regimen of 10–15% oversizing of an endograft based on static CTA images may be inadequate for some patients (27). More severe pulsatility in the aneurysm neck is likely to increase the demand on the fixation and sealing zone of the stent graft. Therefore, data indicate that the preoperative heartbeat-dependent aneurysm neck distension is significantly associated with stent-graft migration after 3 years (7). This could have clinical implications especially in patients with severe hypertension or arrhythmias where the stress on the aortic wall during each cardiac cycle is the highest.

Two phenomena of post-EVAR neck enlargement may be differentiated: an immediate post-implant dilatation, strongly correlated with the percentage of oversize and more likely to reach values equal or higher to 15%, and a subsequent dilatation, relative to the first postoperatively measured diameter, not correlated with the percentage of oversizing but possibly associated with caudad device migration (28). In a recent meta-analysis including 9,721 patients treated with EVAR, 24.6% of cases presented aortic neck dilatation over a follow-up period ranging from 15 to 108 months (29). Furthermore, Tsilimparis et al. have shown that the monthly rate of change for the neck diameter was more rapid in the early postoperative period (30-day period), with an expansion rate of 0.7 ± 0.09 mm/month, and during the third year of follow-up (24–36 months), with a monthly expansion rate of 0.10 ± 0.24 mm (30). However, changes in the aortic neck diameter were statistically significant (*P* < 0.001) only at the 24- to 36-month postoperative interval in this study (30). In the long term, the absence of proximal stent-graft fixation system enhances proximal migration. Conversely, the inter-renal or infrarenal proximal neck dilatation does not depend on the type of proximal fixation but on anatomic factors and on the natural evolution of the aneurysmal disease (31). In the comparative study by Pintoux et al., freedom from dilatation did not differ between infrarenal and suprarenal fixation although proximal graft migration was more frequent with infrarenal fixation (31). Additionally, Oberhuber et al. compared proximal neck dilatation between grafts with different type of fixation, and they found no difference as well (32).

Regarding the type of endovascular devices, some authors have observed neither proximal neck dilatation nor endograft migration with balloon-expandable stents (BESs)—endografts although the endografts were custom-made in these studies (33). In a recent comparative study by Savlovskis et al., repair with certain type of self-expandable stent (SES)—endografts (Nellix; Endologix, Irvine, CA, USA) caused progressive infrarenal aortic neck enlargement although repair with BES endografts did not cause any neck enlargement after a 24–34 months follow-up (34). Additionally, Peirano et al. showed that the diameter reached at initial deployment did not increase further in the long term, which supports the safety and reliability of a modular BES graft and illustrates that this device does not produce dilatation of the proximal neck after deployment (35). However, data on BES grafts are heterogenous as most of the studies have used different types of BES grafts, and no concrete conclusions could be produced. These data suggest that neck enlargement after repair with SES endografts could be related to the force applied by the SES elements and not by the disease progression in the infrarenal neck. However, other studies have concluded that the incidence of neck dilatation is not significantly different among graft types (36). When the endograft is positioned correctly below the renal arteries, there is a high probability of no neck enlargement (37).

Regarding the newer fenestrated grafts, data are lacking as far as their correlation with proximal neck dilatation is concerned. In a recently published meta-analysis, 763 patients treated with fenestrated grafts for more than 2,000 target vessels were included (38). In this study, almost 1.8% of aneurysm sacs were enlarged in size and only 3% of cases presented with graft migration. Fenestrated devices are deployed and fixed at a higher level at the abdominal aorta, above the renal arteries. According to experimental data, aortic wall of AAAs is characterized by degradation of elastic fibers and an increase of collagen/elastin ratio (39). Therefore, given that proximal aorta has a higher concentration of elastin fibers compared to the distal aorta, neck degradation should theoretically be slower than in conventional EVAR patients.

Several risk factors have been identified for contributing to proximal endoleaks or device migration including the type of fixation, initial proximal fixation length, and dilatation/elongation of the infrarenal aortic neck, or short and calcified necks (40, 41). Moreover, patients with large aneurysms and aortic necks and patients with aortic neck circumferential thrombus are also at high risk for aortic neck enlargement after endoluminal repair of AAAs (42). However, Zarins et al. have shown that initial aortic neck length and diameter, aneurysm size, degree of oversizing, use of proximal cuffs at initial implantation, postoperative endoleak, demographic factors, and comorbid conditions are not predictive of stent-graft migration (43). Although aortic neck seems to become larger for at least 24 months after EVAR, other studies have also concluded that expansion rates of the proximal neck do not have a significant correlation with initial neck size, endograft dimensions, aneurysm size change, presence of endoleak, or attachment system fracture (44, 45). Additionally, no correlation between the dilatation of proximal and distal landing levels is observed (14). Concerning changes in the neck angle between the preoperative condition and the immediate postoperative condition, data show that there is no clear relationship found between only the angle of the neck and proximal stent-graft migration (46, 47). Especially in large AAAs, we have found that there is a 15% increase in neck angulation and a 27% decrease in neck length after EVAR, compared to small AAAs, with no difference in outcome (48). However, we have observed that the incidence of neck angulation in patients undergoing secondary interventions after EVAR is increased (49).

## Progression of Proximal AAA Neck after Open Surgery

Although there is statistically significant evidence of increases in the supra- and infrarenal aortic diameters after conventional AAA repair, data show that mean annual increases tend to be small, and clinically relevant increases of 3 mm or more are observed in only a small proportion of cases (50, 51). After open AAA repair, a diameter increase of 0.48–1.0 mm/year has been reported by most of authors (23, 52). This process of dilatation or elongation seems to be continued for a mean period of 42 months, although it should be underlined that in these studies the preoperative aortic neck diameter was taken as baseline, which may distort the actual degree of neck dilatation. However, Lipski and Ernst have found that residual aortic cuff diameter increased more than 5.0 mm and neck length more than 10.0 mm in a significant number of patients, with potentially serious implications for AAA repair (52).

Disorganization and destruction of normal aortic architecture at the ultrastructural level are associated with decreasing aortic distensibility, with low aortic neck distensibility being associated with proximal aortic dilatation at 2 years postoperatively (53). According to many authors, disease progression may be the most common underlying culprit for proximal false-aneurysm formation (54). However, the continuous suture of the proximal anastomosis between aorta and graft may have a purse-string effect causing tapering of the aortic neck in a technically proper anastomosis, and furthermore, straightening of an angulation of the aortic neck may give the impression of a diameter change. As aforementioned, MMPs could also play an important role in this remodeling process, although they could be affected by certain factors such as diabetes mellitus as well (55). However, no direct association of these factors with neck enlargement after open repair has been established yet.

## Comparison Between Endovascular and Open Repair

Several studies have compared endovascular and open repair as far as the risk for proximal aortic neck enlargement and subsequent complications is concerned. After open AAA repair, the infrarenal neck could continue enlarging in both length and diameter, causing caudal displacement of the proximal suture line and predisposing the development of a pseudoaneurysm (56). A similar phenomenon could occur in patients treated with EVAR, particularly in cases with a low initial deployment of the stent graft, subjecting the uncovered neck to further biomechanical deterioration with dilatation and effective shortening (44). Continuing aortic neck dilatation is reported to occur in up to 43% of patients after open repair whereas the incidence of false-aneurysm formation is 1.3–3% during long-term follow-up. However, neck dilatation has been reported in up to 28% of patients at 2 years and in 59% at 4 years and has been shown to be associated with adverse mid-term outcomes after EVAR (57).

In a recent study by Oberhuber et al., patients treated with EVAR or open repair demonstrated similar increases of aneurysmal neck diameters (58). The rate of aortic increase, neck enlargement as well as the rate of reinterventions did not differ between the two groups. This suggests that aortic neck dilatation may be caused by a natural progression of the disease rather than by deviating therapeutic strategies. However, the cause of dilatation has not been clearly defined yet. The radial force of SES grafts can be safely excluded as a major contributor, because otherwise, aortic neck dilatations would not occur after open repairs. A mechanical stress due to aortic cross-clamping resulting in aortic damage with constant dilatation is unlikely; otherwise, the dilatation would happen only in the infrarenal segment. Therefore, the most probable cause is multifactorial, with a natural progression of the aortic disease.

Regarding potential complications, the sutures in an open repair have the potential to “pull-out” and allow direct leak, leading to the formation of a para-anastomotic pseudoaneurysm, or a true *de novo* aneurysm could be formed with the possibility of subsequent rupture. For EVAR, dilatation could result in a loss of friction, with resultant distal migration, and/or a gap between the irregular vessel wall and the device wall, resulting in a type I endoleak. Failure of the proximal seal after EVAR is seen, however, at a relatively early point during follow-up, compared with open repair (59). Time to migration detection, initial aortic neck length, initial proximal fixation length, initial AAA size, initial aortic neck angle and its change, initial aortic neck diameter, degree of stent-graft oversizing, rate of type II and III endoleaks, and stent-graft main body diameter seem to be no different between patients with and without loss of proximal sealing zone, and do not help predict who will lose proximal fixation (56).

## Heterogeneity of Data

Although the SVS has published certain standards in order to ensure a uniform reporting with regard to clinical and morphologic outcomes after AAA repair (60), compliance with these standards has been poor, and therefore heterogeneity of research data has emerged in the available studies. This consensus proposes two core requirements for AAA neck measurements (60):

- “Changes in aneurysm size should be referenced to those measurements obtained from the first set of postoperative images.”
- “Life-table or Kaplan–Meier analysis should be used to analyze freedom from neck enlargement.”

First, many studies (22, 23, 52) have compared preoperative neck diameter with follow-up measurements, not conforming to the aforementioned recommendations, and potentially biasing the findings. Second, the majority of studies assessing neck morphology after treatment (23, 30, 52, 53, 58) have not used a life-table or Kaplan–Meier analysis as a main method of statistical analysis. Thus, another core requirement of the SVS reporting standards was not fully met. Moreover, significant differences in the methodology for the site at which the neck was measured are also observed. Certain number of studies defined the level of measurement in relation to the proximal stent of the endograft, thereby biasing follow-up measurements in patients presenting device migration (59). Other studies defined the term “infrarenal aortic neck” differently. The term was defined as the segment stretching out from directly below the lowermost renal artery (21, 23, 50), to 5.0 mm (36), 6.0 mm (25), or 7.5 mm (24) distally to the lowermost renal artery.

Finally, it should be underlined that the secure deployment of endografts and the avoidance of graft migration rely not only on the fixation at the most proximal segment of the neck but rather on the full integrity of the proximal landing zone. However, there have not been certain uniform recommendations on how to report the longitudinal extent of the infrarenal neck (57). In a former publication of ours, we have highlighted the usefulness of three-dimensional CTA to determine exact morphology and characteristics of infrarenal aortic neck before AAA repair (61). Perhaps the future recommendations will take into consideration the appliance of more advanced diagnostic techniques in order to minimize false measurements.

## Conclusion

Dilatation of proximal aortic neck seems to occur early after both endovascular and open AAA repair, implicating that this phenomenon is more relevant to the progression of aneurysmal disease itself. However, proper placement of grafts just below the level of renal arteries, as well as the use of BES grafts, could reduce this risk although data are still not concluding. Although there is a significant heterogeneity in measurement methods and definitions within several studies, current evidence raises serious concerns regarding long-term durability of proximal aortic graft fixation. More trials comparing proximal neck’s progression between different types or sizes of grafts as well as compliance with SVS reporting standards are warranted in order to produce safer conclusions.

## Author Contributions

KF: design of study, writing, and critical review. GG: design of study, data collection, and writing. FS and KT: writing and critical review. IT, GK, and CB: data collection. GZ: overall supervision.

## Conflict of Interest Statement

The authors declare that the research was conducted in the absence of any commercial or financial relationships that could be construed as a potential conflict of interest.
